# Effect of silver incorporation on the thermoelectric properties of ITO thin films

**DOI:** 10.1039/d5ra00856e

**Published:** 2025-05-15

**Authors:** Sumayya, Sajid Butt, Muhammad Irfan, Muhammad Abdul Basit, Abdul Faheem Khan, Zaka Ansar

**Affiliations:** a Department of Materials Science and Engineering, Institute of Space Technology Islamabad 44000 Pakistan; b Department of Space Science, Institute of Space Technology Islamabad 44000 Pakistan sajid.butt@ist.edu.pk; c National Center for Physics, Quaid-e-Azam University Islamabad 44000 Pakistan

## Abstract

Indium tin oxide (ITO) has been widely investigated for optoelectronic applications. However, the current study focuses on the thermoelectric aspects of ITO thin films. The thermoelectric transport properties of ITO have been further improved through a facile method of silver (Ag) incorporation into ITO thin films. The Ag incorporation introduces a secondary phase, as inferred from detailed structural characterizations, which leads to the creation of heterogeneous interfaces that help in tuning the thermoelectric properties. These interfaces act as carrier scattering centers which lower the carrier's mobility and result in a simultaneous enhancement of the electrical conductivity and Seebeck coefficient. As a consequence, the power factor reached the highest value of 31.75 μW m^−1^ K^−2^ at 625 K, which is about 100% higher than that of the pure ITO. Furthermore, by merely relying on electronic thermal conductivity, a slightly overestimated *ZT* value of 0.15 has been achieved for the optimized Ag content. The proposed simple and rapid route paves the way to further explore the potential of ITO thin films for thermoelectric applications in smart energy conversion and heating–cooling devices.

## Abstract 2

Schematic of electrical conduction in silver incorporated ITO.

## Graphical abstract

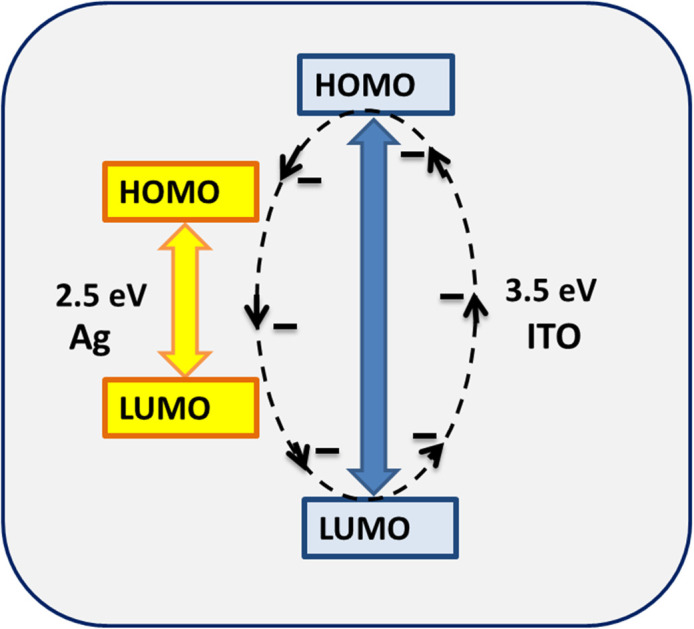

## 1. Introduction

Low-dimensional structures are regarded as the potential means of optimization of thermoelectric performance.^1^ In particular, nanostructuring and heterogeneous thin film formation have received immense importance in the miniaturization of thermoelectric devices.^2–4^ The thin film-based thermoelectric devices are small and lightweight. These two attributes are the basic requirements in the miniaturisation industry such as wearable micro devices.^5^ The thermoelectric efficiency of any device depends upon the performance of each constituent p-type and n-type legs, and is defined by a dimensionless figure-of-merit: *ZT* = (*σS*^2^/*K*)*T*, where *σ*, *S*, *K*, and *T* are the electrical conductivity, Seebeck coefficient, thermal conductivity, and absolute temperature, respectively. Several strategies have been adopted to improve a bulk material's thermoelectric performance, such as band engineering,^6^ modulation of carrier concentration,^7^ elemental doping,^8,9^ spin entropy,^10^ compositing,^11,12^ and nano-structuring.^13,14^ So far, a maximum *ZT* value of 3.1 at 783 K has been achieved for bulk SnSe polycrystalline through hole-doping.^15^ However, the miniaturization of devices has spurred the deployment of thin films in current research. For the thin films, the maximum *ZT* value of ∼2.4 at 300 K has been achieved by p-type superlattice of Bi_2_Te_3_/Sb_2_Te_3_ (ref. 16) but their thermal and chemical instabilities at higher temperatures are to be addressed. There are many strategies have been reported to improve the thermoelectric performance of thin films such as organic–inorganic compositing^4^ and superlattice formation, electron gas (2DEG) formation, magnetic manipulation, and strain engineering.^1^ For transparent thermoelectric applications, n-type, transparent conducting oxides (TCO) are greatly considered due to their high electronic conductivity. Among many TCO, In_2_O_3_ exhibits the highest electrical conductivity (>2000 S cm^−1^)^17^ with a wide band gap of ∼3.7 eV (ref. 18) and has remarkable oxidation resistance and thermal stability as well. The thermoelectric properties of In_2_O_3_ doped with nitrogen (N),^19^ titanium (Ti), zinc (Zn), zirconium (Zr), niobium (Nb), tantalum (Ta) and tin (Sn) have been reported.^1,20–23^ The Sn-doped indium oxide (ITO) showed a *ZT* value of ∼0.28 at 1000 K for the bulk materials.^22^ ITO is considered as favorite material to be used in solar cells, heat mirrors, transparent electrodes, and other displaying devices.^24^ However, the *ZT* for ITO thin films is lower and greatly depends on the deposition conditions, growth methods, and post-deposition treatments.^25–27^ Different methods have been used for the deposition of ITO films such as sol–gel,^28^ e-beam evaporation,^29^ RF sputtering,^26^ DC sputtering,^30^ magnetron sputtering,^25^ ion beam sputtering,^31^ spray pyrolysis,^32^ and pulsed layer deposition (PLD).^33^ Specifically, for ITO, the physical properties are controlled by the concentration of both tin and oxygen vacancies because they help in increasing n-type charge carriers.^34–36^

In this research, we have adopted a facile technique to improve the thermoelectric performance of ITO thin films by the thermal diffusion of Ag. To our knowledge, the previous reports focused especially on deposition techniques but the current work targets post-deposition treatment to further improve thermoelectric performance of ITO thin films.

## 2. Materials and methods

**Fig. 1:**
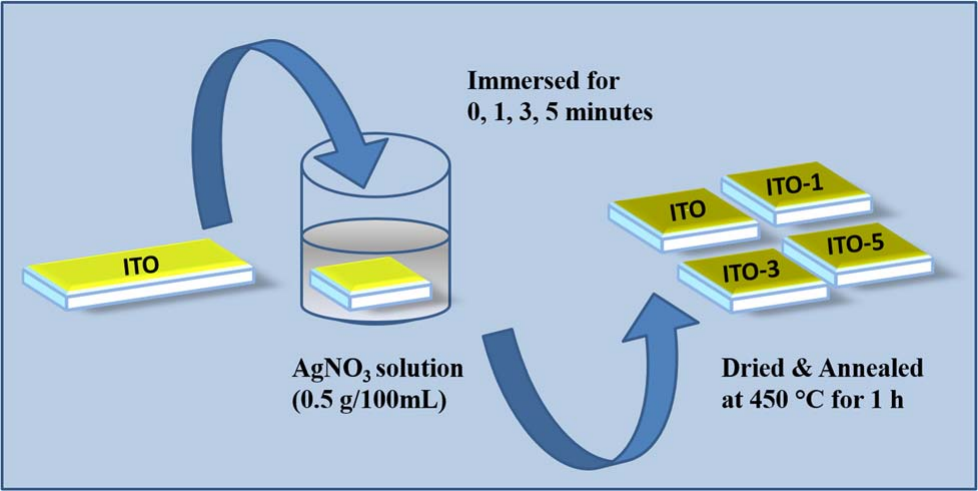
Schematic representation of silver incorporated on ITO films.

ITO thin films having thickness of 100 nm were fabricated over precleaned glass slides through thermal evaporation. The ITO films were immersed in AgNO_3_ solution (0.5 g/100 mL) for different time intervals of 1 minute, 3 minutes, and 5 minutes. After the immersion process, the films were annealed at 450 °C for 1 h. The samples were named according to their immersion time such as ITO, ITO-1, ITO-3, and ITO-5, and the complete experimental design is shown in Fig. 1.

## 3. Characterizations

The obtained set of pure and Ag incorporated ITO films were characterized for chemical, structural, and thermoelectric properties. The chemical composition and binding energies of In, Sn, O, and Ag were determined by X-ray photoelectron spectroscopy (XPS) using the PHI Versa Probe system equipped with Al K_α_ X-rays. The chemical composition was determined by deconvoluting the high-resolution XPS using XPSPEAK41 software. The grain structures and elemental compositions were studied using scanning electron microscopy (FE-SEM, TESCAN-Mira3), coupled with an energy dispersive spectroscopy (EDS) detector. The surface topography and roughness were measured using atomic force microscopy (AFM, Nanosurf FlexAFM).

The electrical conductivity and Seebeck coefficient measurements were done at the Thermoelectric Parameter Test System, Namicro-3L by Joule Yacht. The temperature-dependent charge carrier's concentration (*n*) and mobility (*u*) were measured by the Hall measurement system (ECOPIA HMS 5000).

## 4. Results and discussion

The elemental composition of typical samples like pure ITO and ITO-5 are given in Table 1. The results confirmed the presence of In, Sn, and O in the pure ITO film and In, Sn, O, and Ag for the Ag incorporated ITO-5 sample.

**Table 1:** The chemical composition of ITO and ITO-5

Sample	In (wt%)	Sn (wt%)	O (wt%)	Ag (wt%)
ITO	30.73	13.09	56.18	—
ITO-5	6.98	0.11	89.25	3.67

**Fig. 2:**
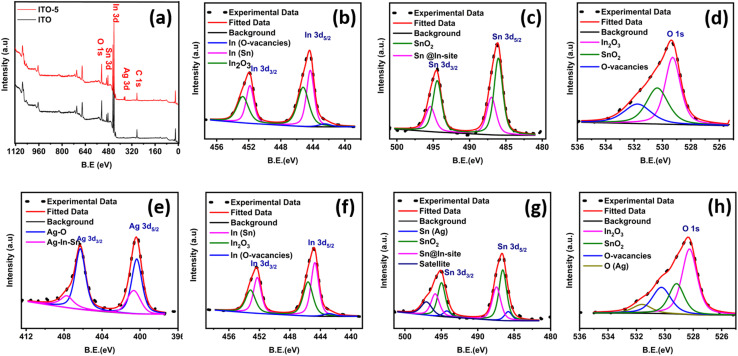
XPS analysis, a full survey scans of ITO and ITO-5 (a). High-resolution spectra of In, Sn and O from ITO (b–d). High-resolution spectra of In, Sn, O and Ag from ITO-5 (e–h).

The chemical composition of typical ITO and ITO-5 samples was studied using XPS data, as shown in Fig. 2. The full survey scan reconfirms the presence of all the necessary elements, as shown in Fig. 2(a). For in depth analysis of the chemical states of the elements in pure ITO and ITO-5 samples, the high-resolution XPS spectrums of O 1s, In 3d, Sn 3d, and Ag 3d were deconvoluted, and a comparative analysis is performed as shown in Fig. 2. The deconvoluted spectrum of all the elements of pure ITO is shown in Fig. 2(b–d), and the ITO-5 is shown in Fig. 2(e–h). The ITO-5 sample indicated the presence of silver, as shown in Fig. 2(e). The peaks observed at binding energies of 444–456 eV in the In 3d spectrum can be assigned to the presence of O vacancies and doping of Sn in the lattice of In_2_O_3_, for ITO and ITO-5 as shown in Fig. 2(b) and (f), respectively. The peaks observed at binding energies of 382–498 eV in the Sn 3d spectrum can be assigned to the presence of SnO_2_ and doping of Sn in the lattice of In_2_O_3_, for ITO and ITO-5 as shown in Fig. 2(c) and (g), respectively. Similarly, the peaks observed at binding energies of 526–534 eV in the O 1s spectrum can confirm the association of O with Sn and In Ag and the presence of O vacancies, as shown in Fig. 2(d) and (h), respectively. For Fig. 2(h), an additional peak at 407.3 eV corresponding to AgO_2_ can be observed which signals the oxidation of silver particles at the surface during annealing. Secondly, Ag incorporation has suppressed O vacancies as inferred by the O-vacancies peaks in Fig. 2(d and h).

**Fig. 3:**
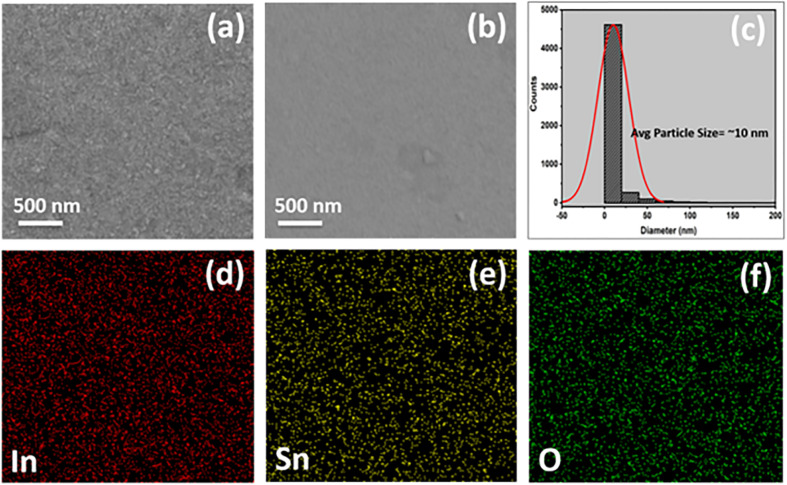
SEM micrographs of pure ITO (a) secondary electron (SE) image (b) backscattered electron (BSE) image (c) particle size distribution estimated by ImageJ software (d–f) elemental distribution of indium (In), tin (Sn) and oxygen (O) images by energy dispersive spectroscopy (EDS).

For the typical samples of ITO and ITO-5, the grain structure and presence of different phases were analyzed using secondary electron (SE) and backscattered electron (BSE) images, respectively. The SE image, as shown in Fig. 3(a), confirms the uniform, densely packed, and voids free films. However, the BSE image in Fig. 3(b), confirms a single-phase system in ITO thin films on a glass substrate because the indium and tin form a solid solution with each other. Fig. 3(c), shows the particle size distribution computed through Image J software along with the average particle size of 10 nm. Fig. 3(d–f) shows the uniform distribution of elements In, Sn, and O further confirming single phase ITO thin film.

**Fig. 4:**
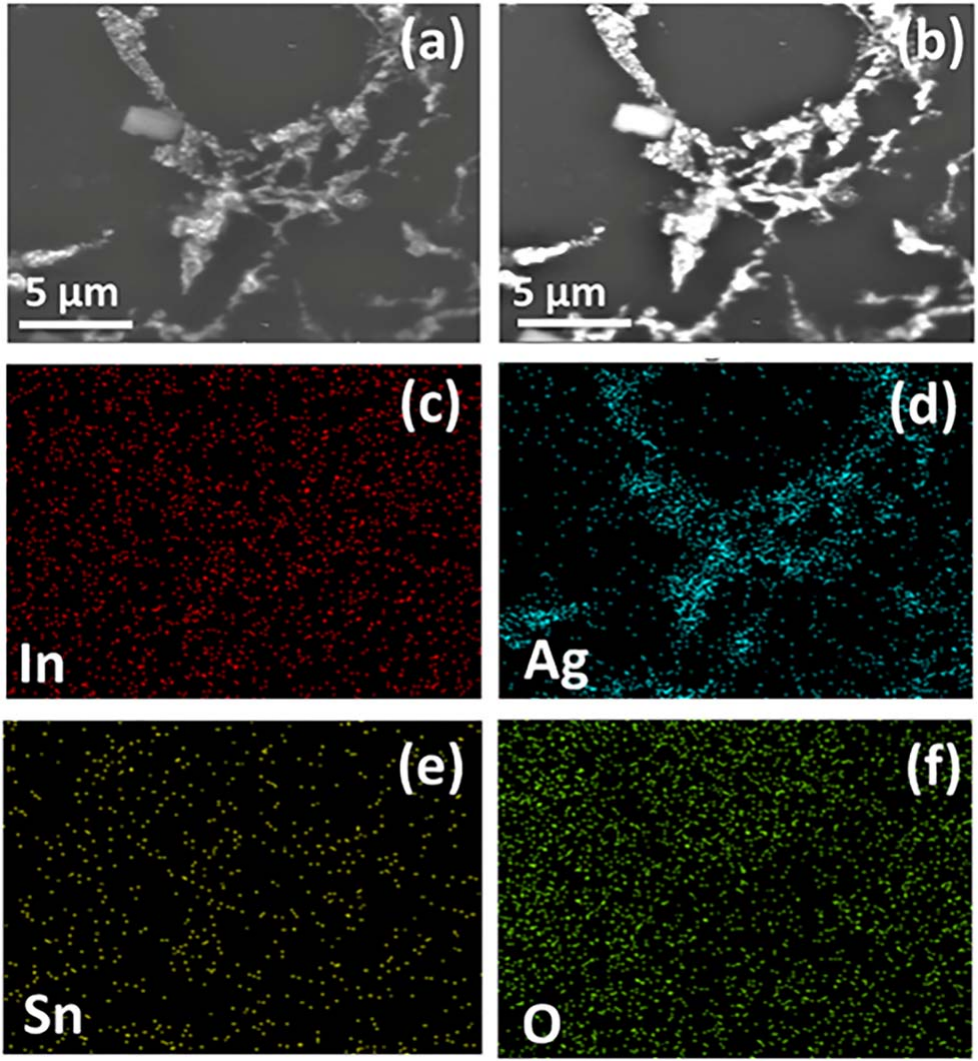
SEM micrographs of silver incorporated ITO for 5 minutes (ITO-5) (a) secondary electron (SE) image (b) backscattered electron (BSE) image (c–f) elemental distribution of indium (In), silver (Ag), tin (Sn) and oxygen (O) images by energy dispersive spectroscopy (EDS).

SE and BSE based images for the ITO-5 sample are given in Fig. 4(a and b), respectively. The bright contrast regions clearly indicate the presence of Ag clusters in ITO thin films. This Ag clustering acts as a bridging among the grains of ITO resulting in an increased electrical conductivity. The elemental mapping for ITO-5 is shown in Fig. 4(c–f), in which the presence of Ag clustering can further be confirmed as shown by Fig. 4(d).

**Fig. 5:**
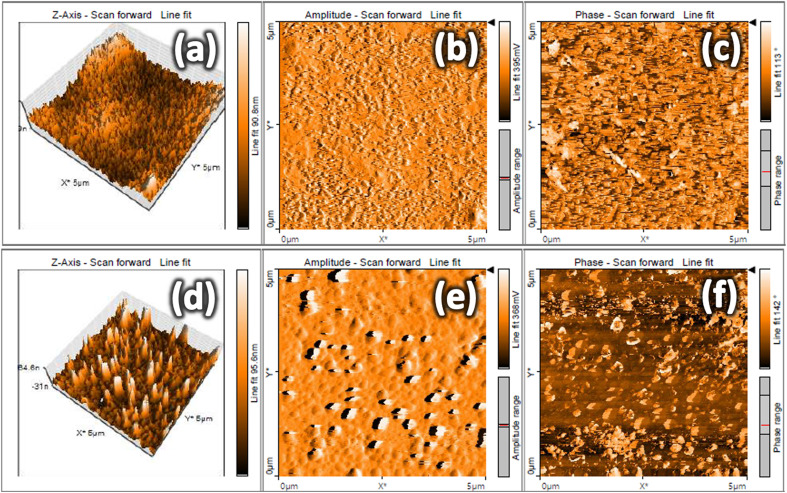
Atomic force microscope (AFM) images of pure ITO (a–c) and silver incorporated ITO for 5 minutes (ITO-5) samples (d–f).

The surface topography of ITO and ITO-5 at a scale of 5 μm × 5 μm is shown in Fig. 5. For the pure ITO uniform surface features are observed as shown in Fig. 5(a–c). However, for the ITO-5 sample, some bigger grains can be observed over the surfaces, as shown in Fig. 5(d–f), which are predominately the Ag grains, which could be identified in SEM images as well. The pure ITO and silver incorporated ITO films appeared dense and without any detectable voids which facilitates conduction of charge carriers.

**Fig. 6:**
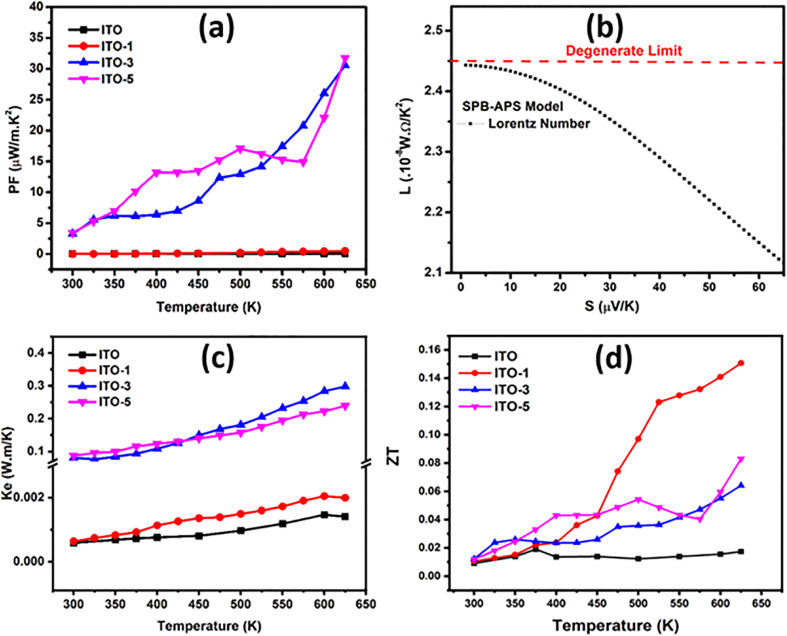
Thermoelectric transport measurements of pure ITO and silver incorporated ITO with variable time intervals (ITO-1, ITO-3, and ITO-5) (a) electrical conductivity *vs.* temperature (b) carrier concentration *vs.* carrier mobility (c) activation energy (d) Seebeck coefficient *vs.* temperature.

The temperature dependent thermoelectric transport measurements of all the series of pure and silver incorporated ITO films are shown in Fig. 6. The electrical conductivity of all the prepared samples is shown in Fig. 6(a). The graph of electrical conductivity *vs.* temperature depicts three results. (1) The electrical conductivity of all the samples is directly related to temperature, inferring semiconducting behavior. (2) The electrical conductivity of all the Ag-incorporated samples increases by increasing Ag incorporation time. (3) The electrical conductivity increases for samples immersed for 1 min (ITO-1), 2 min (ITO-2) and 3 min (ITO-3). This increase is associated with the higher intrinsic conductivity of Ag, which bridges among ITO grains. Secondly, the oxygen vacancies are created at higher temperatures.^27^ In the case of pure ITO an abrupt increase in electrical conductivity is observed at 450 K, this abrupt increase is due to the phase transition of ITO films from amorphous to polycrystalline phases.^37^ The incorporated silver provides the conductive channels in the ITO films and as a result, electrical conductivity increases, and a maximum electrical conductivity of 20 699.43 S m^−1^ is achieved by ITO-3 at 625 K. The increase in electrical conductivity is also associated with the increase in immersion time but up to the saturation limit of silver^38^ in ITO at specific temperatures. In this research, the silver incorporated ITO for five minutes showed a maximum electrical conductivity of 12 012.80 S m^−1^ at 300 K which increases till 430 K and then decreases as compared with the electrical conductivity of ITO-3. After 430 K, the carrier's scattering increases with temperature which decreases the carrier's mobility and as a result, the electrical conductivity of ITO-5 decreases as compared with ITO-3 at higher temperatures. The charge carrier concentration *vs.* carrier's mobility of ITO and ITO-5 is shown in Fig. 6(b). The carrier's concentration increases with the incorporation of silver but the mobility decreases. The obtained results are in good agreement with the similar studies conducted over CdS thin films incorporated with Ag^9^ and In.^39^

The modified Arrhenius plots are shown in Fig. 6(c). The linearly fitted curves showed two regions *i.e.*, low temperature (300 K to 425 K) and high temperature (425 K to 625 K) region. The activation energy (*E*_a_) was calculated from the slopes of the linearly fitted curves and named *E*_a1_ for low temperature region and *E*_a2_ for high temperature region, respectively. The calculated *E*_a1_ and *E*_a2_ are tabulated in Table 2. The *E*_a1_ is lower than *E*_a2_ because at higher temperature higher activation energy is required for the conduction mechanism. For pure ITO *E*_a1_ < *E*_a2_ due to the phase transition from the amorphous phase to the polycrystalline phase.^37^ At higher temperatures, with the increase in immersion time *E*_a2_ increases and reaches to maximum of 103.3 meV in ITO-5 because the carrier scattering increases with the temperature and ITO-5 with maximum carriers showed the highest *E*_a2_.

**Table 2:** The activation energies of pure ITO and silver incorporated ITO associated with low temperature region from 300 K to 425 K (*E*_a1_) and high temperature region from 425 K to 625 K (*E*_a2_)

Sr. no.	Sample ID	*E*_a1_ (meV)	*E*_a2_ (meV)
1	ITO	25.43	85.2
2	ITO-1	62.25	68.55
3	ITO-3	39.59	74.3
4	ITO-5	41.55	103.3

The Seebeck coefficient (*S*) trend towards increasing temperature is shown in Fig. 6(d). The entire sample series showed negative *S* which proves the n-type conduction mechanism, and the majority charge carriers are electrons in pure as well as in Ag incorporated ITO films. The maximum *S* of −20.45 μV K^−1^ at 625 K was observed by pure ITO and it was improved to −55.57 μV K^−1^ for ITO-5 at 625 K, which is about 171% larger than that of the pure ITO films. In ITO-1, the effective conductive channel provided by silver improved the mobility of carriers with the increase in temperature.^40^ The incorporation of silver in ITO also formed a secondary phase which is also evident from the BSE image of ITO-5 captured by SEM as shown in Fig. 4(a). The secondary phase creates the heterogeneous interfaces and grain boundaries as a result these defects act as carriers scattering centers which lowered the carriers mobility and improved the Seebeck coefficient.^41^ Moreover, the energy filtering effect^42^ and the establishment of local thermal gradient^43^ are other reasons that helped in improving the Seebeck coefficient. However, as the silver content increases as in ITO-3 and ITO-5, the increased carrier's concentration for the dominating factor reduces *S*.

**Fig. 7:**
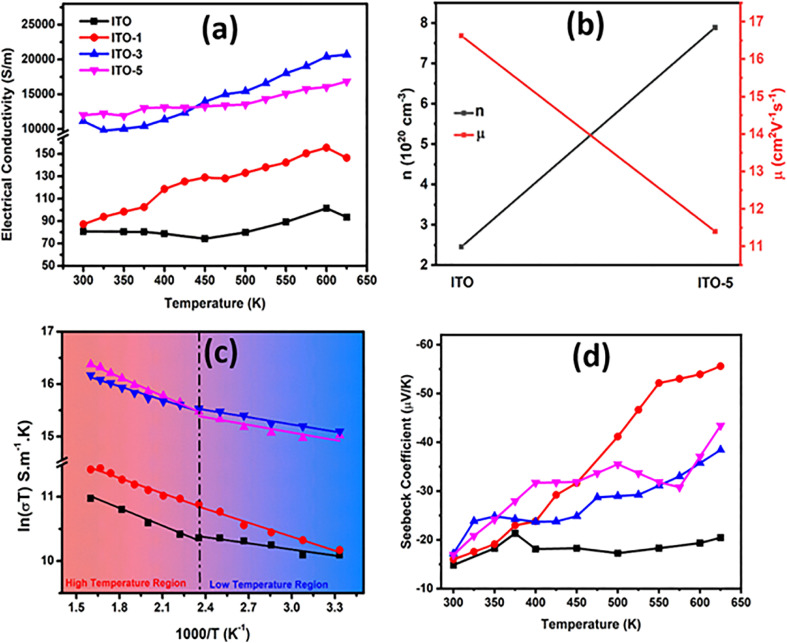
Thermoelectric transport measurements of pure ITO and silver incorporated ITO with variable time intervals (ITO-1, ITO-3, and ITO-5) (a) power factor *vs.* temperature (b) Lorentz number *vs.* Seebeck coefficient (c) electronic thermal conductivity *vs.* temperature (d) figure of merit (*ZT*) *vs.* temperature.

Fig. 7(a) shows the calculated power factor (PF) *vs.* temperature. The PF was calculated by the product of the square of the Seebeck coefficient and electrical conductivity. The highest power factor of 31.75 μW m^−1^ K^−2^ was attained by ITO-5 at 625 K. Thermal conductivity is a crucial parameter for the determination of the thermoelectric performance of any material. The total thermal conductivity is the sum of the electronic (*K*_e_) and the lattice thermal conductivity (*K*_L_). In thin films, the lattice thermal conductivity is difficult to measure^3,44^ due to the complex interplay of nanoscale physics and the limited availability of measurement facilities. Thus, *K*_L_ was ignored and only *K*_e_ was employed for *ZT* calculation. *K*_e_ was calculated using Wiedemann–Franz law; *K*_e_ = *LσT*, where, *L* is the Lorenz as shown in Fig. 7(b), determined using the constraints of the semi-parabolic band (SPB) coupled with acoustic phonon scattering (APS).^45^ The *K*_e_ follows the same trend as electrical conductivity, as shown in Fig. 7(b). The resultant *ZT* value is overestimated, as shown in Fig. 7(c). The highest *ZT* of 0.15 at 625 K has been achieved ITO-1, which is about 100 larger than that of the pristine ITO sample.

## 5. Conclusion

A rapid technique enabled swift silver incorporation in ITO thin films demonstrating significant improvements in thermoelectric properties. The optimized thermoelectric parameters were observed for ITO samples incorporated with Ag for different immersion times. The ITO-3 composition showed the highest electrical conductivity of 20 699.43 S m^−1^ at 625 K, while the ITO-1 revealed the maximum Seebeck coefficient of −55.57 μV K^−1^, the ITO-5 exhibited the highest power factor of 31.75 μW m^−1^ K^−2^ at the same temperature. The remarkable enhancement in power factor was observed, increasing from 0.039 μW m^−1^ K^−2^ for the pure ITO to 31.75 μW m^−1^ K^−2^ for the ITO-5 sample, at 625 K. Overall, the ITO-1 displayed the highest *ZT* of 0.15 which is about 100% larger than that of the pristine ITO. This substantial improvement is credited to the incorporated Ag clusters in the matrix of ITO, altering the charge carrier concentration and mobility and the temperature induced formation of defects, which collectively leads to enhanced thermoelectric properties. Our research reveals a facile and rapid route to improve the thermoelectric properties of ITO thin films.

## Data availability

All the data are available in the manuscript in the detail. Further information, if required will be provided.

## Conflicts of interest

All the authors declare that there are no conflicts of interest.
